# P309: Knowledge and occupational exposure to blood and body fluids among medical students

**DOI:** 10.1186/2047-2994-2-S1-P309

**Published:** 2013-06-20

**Authors:** L Markovic-Denic

**Affiliations:** 1 Institute of Epidemiology, Faculty of Medicine, University of Belgrade, Belgrade, Serbia

## Introduction

Medical students, as other health care workers, are at risk for occupational needlestick injuries (NSIs) which can result in exposure to blood-borne pathogens and substantial health consequences.

## Objectives

To determine the frequency of NSIs and the knowledge, attitudes and perception of risks of blood-borne diseases of the medical students.

## Methods

The cross-sectional study was conducted among four and sixth–year students of the Faculty of Medicine, Belgrade. Data were obtained using a self-reported anonymous questionnaire whichincluded social-demographic data, NSIs, level of knowledge, risk-awareness, reporting behavior and vaccination status against hepatitis B.

## Results

The questionnaire was filled-in and returned by 80.9% of fourth-year and 63.1% of sixth-year medical students. The study group included more female students ( 69.3% in fourth and 67,1% in final year). Out of all, 8.4% fourth and 9.8% sixth-years students had at least one accident (p>0.05).The largest number of accidents occurred during blood-taking practices. The accident was not reported at all by 43.3% students. Overall, final year medical students were significantly more knowledgeable regarding sharps injuries than the fourth year (p<0.01). Only 25.9% of all students, without differences according to year of studing, were vaccinated against hepatitis B, nevertheless that vaccine is free for them. About two-tiers of students recognized the risk of hepatitis B transmission.

## Conclusion

This study demonstrated that intensive education which can improve the knowledge about the transmission of bloodborne infections and reduce the number of needlestick injuries of medical students must be implemented.

## Disclosure of interest

None declared.

